# Changes in the attractiveness of medical careers and career determinants during the bachelor’s program at Zurich medical schools

**DOI:** 10.1186/s12909-024-05693-8

**Published:** 2024-06-26

**Authors:** Katja Weiss, Stefania Di Gangi, Markus Inauen, Oliver Senn, Stefan Markun

**Affiliations:** Institute of Primary Care, University of Zurich and University Hospital of Zurich, Pestalozzistrasse 24, Zurich, 8091 Switzerland

**Keywords:** Undergraduate medical education, Student perception, Career choice, Medical specialties

## Abstract

**Background:**

Monitoring the career intentions of medical students during their undergraduate studies could help to address the shortage of physicians, particularly in general practice. This study aimed to investigate changes in medical students' career openness, attractiveness and determinants of medical career choice during their bachelor’s studies.

**Methods:**

The design was cross-sectional, recruiting all medical students who started a bachelor’s program in one of the four different educational tracks in Zurich, Switzerland, in the fall of 2019 (first survey) and completed it in the summer of 2022 (second survey). Students’ perceptions of the attractiveness and determinants of different medical career options were assessed using a structured online questionnaire. Absolute changes between the two-time points were reported in percentage points overall and by educational track. Regression analysis was used to examine the association of student characteristics and determinants of career options with the attractiveness of each option.

**Results:**

We surveyed 354 medical students at the beginning and 433 at the end of the bachelor’s program (participation rate: 71.1% and 86.9%, respectively). Overall, the proportion of students open to all proposed medical career options decreased (from 52.8% to 43.8%, *p* = 0.004). The attractiveness of outpatient gynecology or pediatrics increased (from 27.4% to 43.4%, *p* < 0.001), whereas the attractiveness of both general and specialized inpatient care decreased (from 47.8% to 40.3%, *p* = 0.05 and from 71.1% to 61.1%, *p* = 0.006 respectively). There was an increase in the proportion of students who perceived part-time work, autonomy and relationships with patients as important career determinants (from 47.3% to 64.7%, *p* < 0.001; from 63.3% to 77.8%, *p* < 0.001; from 80.8% to 89.3%, *p* = 0.002 respectively), while the importance of reputation and career opportunities decreased (from 42.6% to 26.2%, *p* < 0.001; from 79.2% to 63.6%, *p* < 0.001 respectively). The importance of part-time work and relationships with patients were positively associated with the attractiveness of general practice.

**Conclusions:**

During the bachelor’s program, the attractiveness of a career in general practice tended to decrease, but the importance of part-time work, autonomy and relationships with patients as career determinants increased. Helping students understand how these determinants relate to general practice may increase their interest in the profession.

**Trial registration:**

Not applicable.

**Supplementary Information:**

The online version contains supplementary material available at 10.1186/s12909-024-05693-8.

## Background

Medical students’ motivations for choosing their specialty are diverse and influenced by the socioeconomic context in which they live: students from high-income countries are often motivated by scientific and humanitarian reasons, while those from upper-middle and lower middle-income countries are also influenced by societal factors such as prestige or job security [1]. As global health care becomes more complex and medical careers evolve, understanding what makes medical careers attractive during medical education is essential to the maintenance, sustainability and effectiveness of healthcare systems worldwide.

Several factors influence medical students' career choices [2]. These include demographic variables such as age and gender [2], opportunities for entrepreneurship in different practice settings (e.g. employed in a hospital or self-employed in private practice), patient contact, income, academic opportunities [3–6], the medical curriculum as well as exposure to different medical specialties through clinical rotations [7] and the culture of the medical school itself [8]. During their studies, the emphasis on career determinants may shift from intrinsic motivations, such as personal values, self-confidence and a positive attitude towards patient care, to extrinsic factors, including status and reputation, working conditions, and experiences within specific specialties [2]. Gender also plays a role in shaping these motivations, with intrinsic motivations being more common among female students and extrinsic ones among male students [9, 10].

Understanding all of these factors is essential to developing strategies to address local healthcare workforce challenges, which are also affected by the global health worker migration [11]. Primary care workforces are declining globally [12, 13]. In Switzerland, this decline is of particular concern, as one in four physicians is 60 years of age or older and expected to retire within a few years, exacerbating the current workforce shortage [14]. General practitioners (GPs), who provide primary care, play a key role in most healthcare systems, making a shortage of GPs particularly problematic [15, 16]. In Switzerland, as in many other countries, GPs are the first point of contact for patients and act as coordinators in the healthcare system. The majority of the Swiss population has a health insurance model that places GPs in a gatekeeping role and thus in an even more central position [17]. Unfortunately, it is also predicted that the GP workforce will continue to fall short of demand, as the number of GPs and pediatricians in Switzerland is expected to decrease by 8.4% by 2030 [18–20].

Therefore, to ensure that medical education adapts and meets the evolving needs of society and the healthcare system, it is necessary to make general practice a more attractive career choice for medical students [21].

Swiss medical education consists of two main levels: the bachelor's degree in Human Medicine and the master's degree in Human Medicine, both having a duration of three years. While the bachelor’s degree program focuses on a solid foundation in basic medical sciences and includes only introductory clinical skills training, the master’s degree focuses on clinical training, including rotations in various medical specialties. Swiss medical students are assigned to specific medical schools through a centralized and competitive admissions process designed to select candidates who are likely to complete medical school and to take into account regional distribution.

Given the dynamics of the medical workforce and societal demands, medical students’ career interests and intentions require monitoring. Therefore the present study, conducted at the University of Zurich, Switzerland, aimed to answer the following questions:

1) Do the career openness and attractiveness of different medical career options change during the bachelor’s program in medical education?

2) Does the importance of career determinants change during the bachelor’s program in medical education?

3) Which factors and career determinants are associated with the attractiveness of each medical career option?

Specifically, we aimed to evaluate student-perceived attractiveness of individual career options with a particular focus on general practice in comparison to other specialties. Previous studies have examined the stability of medical students’ career interests using two measures, typically at entry and exit of medical school [22–24]. With this study, we expected to provide further insights into aspects that should be considered in specific interventions in medical education to increase interest in general practice.

## Methods

### Design and participants

The study consisted of two rounds of a cross-sectional survey at the beginning and end of the bachelor's program, conducted in fall 2019 and summer 2022, among students enrolled in different medical education tracks at the University of Zurich: the largest track includes both a bachelor’s and a master’s degree and teaches medicine without an explicit teaching focus (Med_General_); two other tracks are bachelor’s programs that begin at the University of Zurich and continue at the University of Lucerne or St. Gallen for the master's degree, with a focus on primary care (Med_PrimCare_). An additional track is located at the Swiss Federal Institute of Technology and is a bachelor’s program followed by a master's program at the University of Basel, Lugano or Zurich, with a stronger focus on research and technology (Med_ResTech_) [25, 26]. The same questionnaire was used in both rounds and was specifically designed for the purposes of this study. The first round took place during a plenary lecture. Methods, definitions and results of the first survey have been published [27]. Since attendance at lectures was lower in the third year, the second round was conducted by inviting students via email or chat groups, with a reminder email and a raffle among survey participants (prizes were 10 vouchers valued at CHF 100 each to spend in a store chosen by the winner).

### Questionnaire

The questionnaire at both time points was in German and included items measuring the attractiveness of different medical career options and the importance of determinants of career choice. An English translation of the survey is available [27]. The attractiveness of seven proposed specific or aggregated medical career options (general practice, outpatient gynecology/pediatrics, specialized outpatient care other than gynecology/pediatrics, inpatient general internal medicine, specialized inpatient care, academic, medical technology industry) was measured on a five-point Likert scale (from “excluded goal” to “the only goal”). Similarly, a five-point Likert scale (from “not at all important” to “very important”) was used to rate the perceived importance of eight career determinants (relationship with patients, primarily performing medical activities, autonomy, part-time work, career opportunities, income, reputation, political context) which have been identified in the literature as potentially important [2–6]. The questionnaire also included information on the medical education track attended, demographic characteristics (place of residence, gender, age) and the score obtained in the mandatory and selective aptitude test [27] upon entry to the medical education program.

### Ethics

This study did not fall within the scope of the Swiss Human Research Act, as no health-related data were used [28] and was therefore not subject to approval by the local ethical committee. Approval was obtained from the respective universities. Participation in the survey was voluntary and participants were informed and agreed that their data would be collected, summarized and published anonymously.

### Study outcomes

Study outcomes were changes during the bachelor’s program in the percentage of students who: 1) perceived complete openness to all proposed medical career options; 2) perceived a proposed medical career option as attractive; 3) rated a proposed determinant of career choice as important. Outcomes were assessed both in the total population and within education tracks. Furthermore, we assessed the association of student characteristics and the perceived importance of career determinants with the attractiveness of specific career options.

In terms of career openness, respondents were categorized as being: “completely open” to all proposed career options if they did not rate any of the proposed career options as ‘the only goal’ or as ‘an excluded goal’; “committed” if they rated any of the proposed career options as “the only goal”; “partially open” if they did not rate any career option as “the only goal” but at least one as “excluded goal”. Regarding the attractiveness of career options, respondents were categorized as perceiving a proposed career as “attractive” if they rated the career as “rather attractive” or “the only goal”. Regarding the importance of the determinants of career choice, respondents were categorized as perceiving a proposed determinant as “important” if they rated it as “rather important” or “very important”.

### Statistical analysis

Statistical analysis was performed using the R software (version 4.1.0) [29]. Student characteristics and measurements at the two-time points were described as numbers and percentages, n(%), for categorical or binary variables and as mean (standard deviation (SD)) or median [interquartile range (IQR)], as appropriate, for continuous variables. Available case analysis was performed and the number of non-missing observations was reported. For group comparisons between the two time points, chi-squared test was used for categorical or binary variables and t-test or Wilcoxon test, as appropriate, for continuous variables. The attractiveness of medical career options and the importance of career determinants as a five-point Likert scale at the two-time points were presented graphically using a diverging bar chart. Study outcomes 2) and 3), as described above, were presented using a dumbbell plot, also known as a connected dot plot, with additional reporting of absolute differences with 95% confidence interval and *p*-value from chi-square test. For all analyses, students from the two Med_PrimCare_ tracks were combined into a single track, as they both focused on aspects of primary care. Logistic regression analysis, multivariable and univariable, was carried out to identify student characteristics and career determinants associated with the attractiveness of each medical career option. An additional multivariable logistic analysis was carried out to examine the association between student characteristics and the importance of career determinants. The selection of variables for multivariable models was based on a stepwise backward approach, starting from a full model including all variables and excluding them using the Akaike information criterion (AIC). The results of regression analyses were reported as odds ratios (OR) with 95% confidence intervals. The final model results were presented in a forest plot. Due to the pre-specified number of observed cases, no power calculation was performed. A *p* ≤ 0.05 was used to determine statistical significance.

## Results

### Population characteristics

In the first-year survey, a total of 354 medical students participated (Med_General_ 201, Med_PrimCare_ 59, Med_ResTech_ 94) and the overall participation rate was 71.1% (participation rates 67.0%, 67.0% and 85.5% respectively). In the third year, 433 medical students participated (Med_General_ 269, Med_PrimCare_ 83, Med_ResTech_ 81) and the overall participation rate was 86.9% (Med_General_ 89.7%, Med_PrimCare_ 94.3%, Med_ResTech_ 73.6%). Participants were predominantly female, with *n* = 199 (64.8%) in the first year and *n* = 281 (66.0%) in the third year, with a mean age of 20.2 years (SD = 2.2) in the first year and 22.9 years (SD = 2.2) in the third year. All student characteristics are described in Table 1.

**Table 1:** Students’ characteristic and career openness. Number of non-missing observations (n) are reported when different from the sample size (N)

		First year	Third year	*p*-value
N		354	433	
Sex n(%)	female	199 (64.8)	281 (66.0)	0.809
	male	108 (35.2)	145 (34.0)	
		*n* = 307	*n* = 426	
Age (mean (SD))		20.21 (2.24)	22.93 (2.23)	< 0.001
		*n* = 298	*n* = 421	
Medical education track	Med_General_	201 (56.8)	269 (62.1)	0.030
	Med_ResTech_	94 (26.6)	81 (18.7)	
	Med_PrimCare_	59 (16.7)	83 (19.2)	
Assignment concordant with application^a^ n(%)	no	49 (13.8)	60 (13.9)	1.000
	yes	305 (86.2)	373 (86.1)	
Aptitude test score (median (IQR))		89.00 [80.25, 95.75] *n* = 290	91.00 [83.75, 96.00] *n* = 320	0.091
Domicile in medical education track canton n(%)	no	163 (46.0)	139 (32.1)	< 0.001
	yes	191 (54.0)	294 (67.9)	
Career openness				
Overall n(%)	completely open	168 (52.8)	188 (43.8)	0.004
	partially open	96 (30.2)	180 (42.0)	
	committed	54 (17.0)	61 (14.2)	
		*n* = 318	*n* = 429	
Med_General_ track n(%)	completely open	96 (56.1)	111 (41.6)	0.002
	partially open	52 (30.4)	126 (47.2)	
	committed	23 (13.5)	30 (11.2)	
		*n* = 171	*n* = 267	
Med_ResTech_ track n(%)	completely open	51 (54.8)	33 (41.2)	0.057
	partially open	17 (18.3)	27 (33.8)	
	committed	25 (26.9)	20 (25.0)	
		*n* = 93	*n* = 80	
Med_PrimCare_ track n(%)	completely open	21 (38.9)	44 (53.7)	0.134
	partially open	27 (50.0)	27 (32.9)	
	committed	6 (11.1)	11 (13.4)	
		*n* = 54	*n* = 82	

^a^Case in which the student was assigned to the educational track applied for

### Changes in career openness

The percentage of students who perceived themselves as completely open to all proposed career options decreased from 52.8% in the first year to 43.8% in the third year, but the percentage of students who perceived themselves as committed to a career also decreased from 17.0% to 14.2%, *p* = 0.004. The same trend was observed in the Med_General_ track, where openness decreased from 56.1% to 41.6% and commitment from 13.5% to 11.2%, *p* = 0.002 (Table 1).

### Changes in the attractiveness of career options

**Fig. 1:**
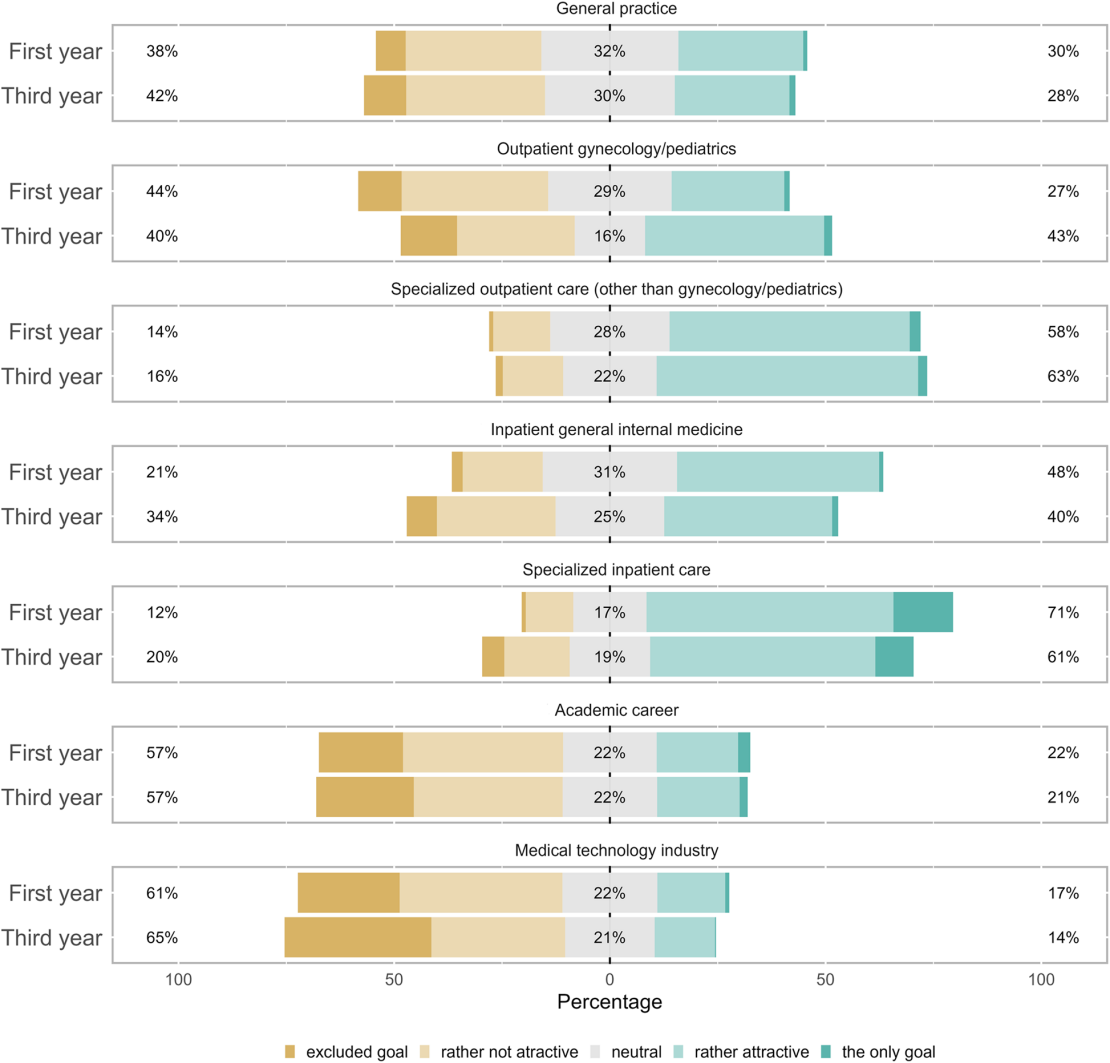
Attractiveness of medical career options during the bachelor’s program. Survey results (Likert-scale) at the beginning (first year) and at the end (third year) of medical school (bachelor’s program). The right side shows the percentages of positive responses (rather attractive/the only goal). In the middle are the percentages of neutral responses, and on the left are the percentages of negative responses (excluded goal/not very attractive)

**Fig. 2:**
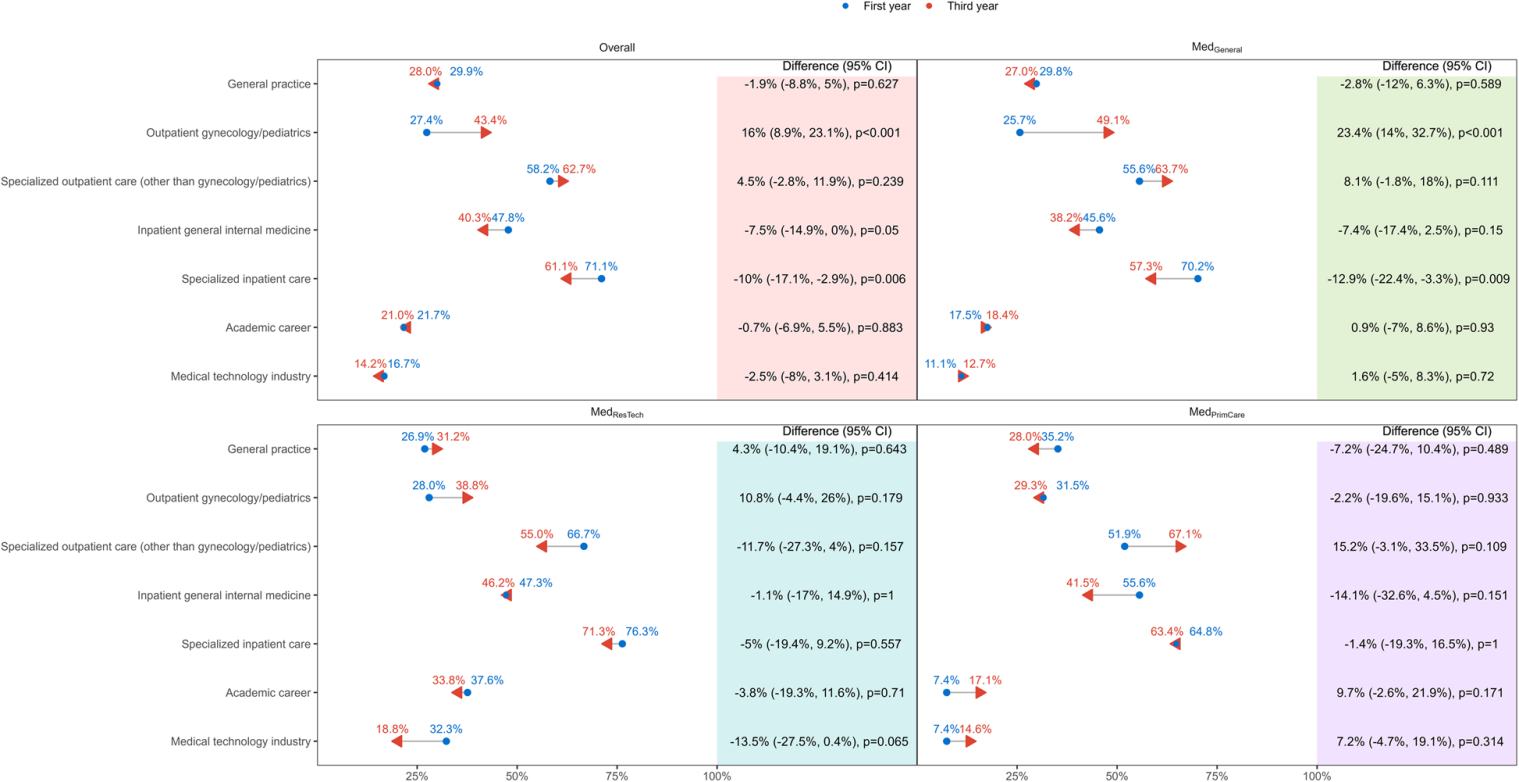
Changes in the attractiveness of medical career options during the bachelor’s program. Absolute differences, overall and by track, in the percentage of medical students attracted to each specialty at the two time-points, at the beginning (first year) and at the end (third year) of the bachelor’s program, are reported with 95% confidence interval and *p*-values. Third-year observations are indicated by arrowheads to highlight the direction. CI: confidence interval; Med_General_: medical education track without specific focus; Med_PrimCare_: medical education track with a focus on primary care; Med_ResTech_ medical education track with a focus on research and technology

There were significant changes during the bachelor’s program in the attractiveness of three of the seven proposed career options (see Fig. 1, results in Likert scales, and Fig. 2, results in percentages and absolute differences between the two-time points, overall and at the level of the medical education tracks). A strong increase in attractiveness was observed in outpatient gynecology/pediatrics (from 27.4% in the first year to 43.4% in the third year, *p* < 0.001). At the same time, the attractiveness of inpatient care disciplines decreased significantly (inpatient general internal medicine: from 47.8% to 40.3%, *p* = 0.05; specialized inpatient care: from 71.1% to 61.1%, *p* = 0.006). Nevertheless, specialized medical career options, both outpatient and inpatient, continued to outrank inpatient general internal medicine or general practice. The attractiveness of general practice did not change. We observed, in addition, that among the students who found general practice attractive, the proportion of students who also found other specialties attractive increased during the bachelor’s program.

### Changes in determinants of career choice

**Fig. 3:**
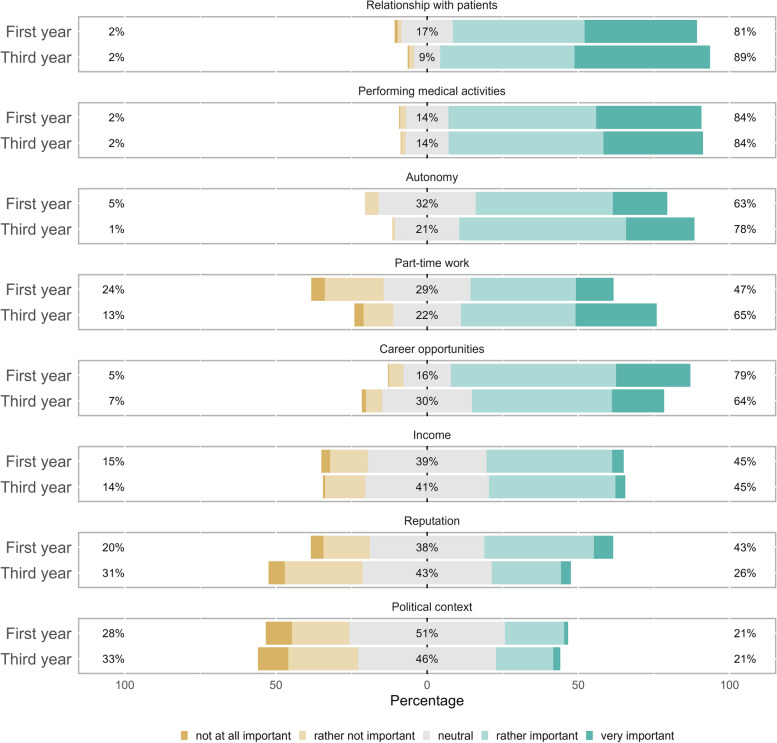
Importance of career determinants during the bachelor’s program. Survey results (Likert-scale) at the beginning (first year) and at the end (third year) of medical school (bachelor’s program). The right side shows the percentages of positive responses (rather important/very important). In the middle are the percentages of neutral responses, and on the left are the percentages of negative responses (not at all important/rather not important)

**Fig. 4:**
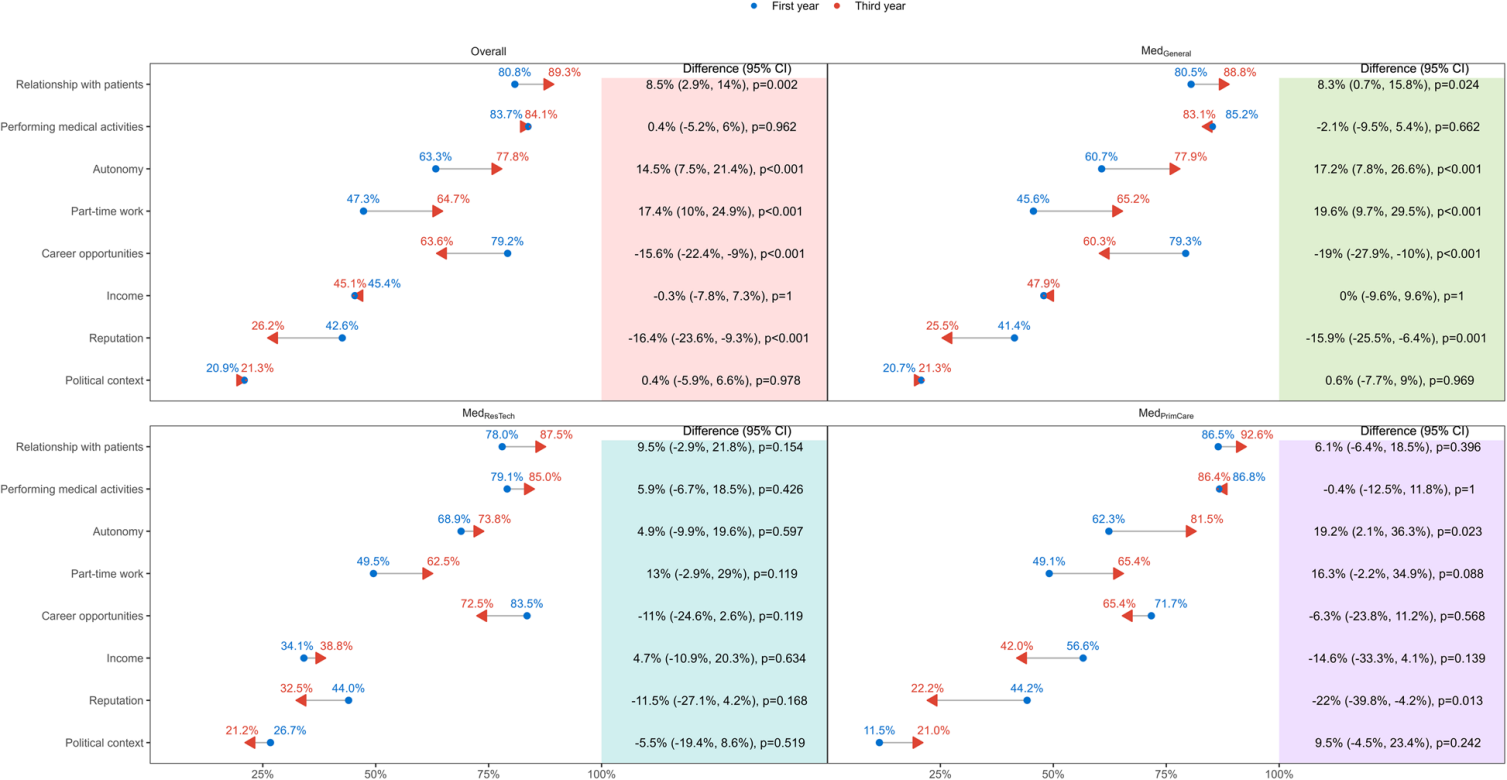
Changes in the importance of career determinants during the bachelor’s program. Absolute differences, overall and by track, in the percentage of medical students rating each factor as important at both time points, at the beginning (first year) and at the end (third year) of the bachelor’s program, are reported with 95% confidence intervals and *p*-values. Third-year observations are indicated by arrowheads to highlight the direction. CI: confidence interval; Med_General_: medical education track without specific focus; Med_PrimCare_: medical education track with a focus on primary care; Med_ResTech_ medical education track with a focus on research and technology

There were significant changes during the bachelor’s program in five of the eight proposed determinants of career choice (see Fig. 3, results in Likert scales, and Fig. 4, results in percentages and absolute differences between the two-time points, overall and at the level of the medical education tracks). The largest change was an increase in the perceived importance of part-time work (from 47.3% in the first year to 64.7% in the third year, *p* < 0.001), followed by an increase in the importance of autonomy (from 63.3% to 77.8%, *p* < 0.001) and the relationship with patients (from 80.8% to. 89.3%, *p* = 0.002). On the other hand, there was a decrease in the importance of reputation (from 42.6% to 26.2%, *p* < 0.001) and career opportunities (from 79.2% to 63.6%, *p* < 0.001). These trends were confirmed by multivariable analysis, which also adjusted for sex and medical education track (Additional file 1 Table 1). At the level of medical education tracks, all these trends were observed in the Med_General_ track and only the trends for autonomy and reputation in the Med_PrimCare_ track, although they were not confirmed by multivariable analysis. There were no statistically significant changes in the career determinants within the Med_ResTech_ track. In addition, after correcting for time effect and medical education track, male students were more likely than female students to value reputation and career opportunities, but less likely to rate part-time work and relationships with patients as important determinants of career choice.

### Factors associated with the attractiveness of medical career options

**Fig. 5:**
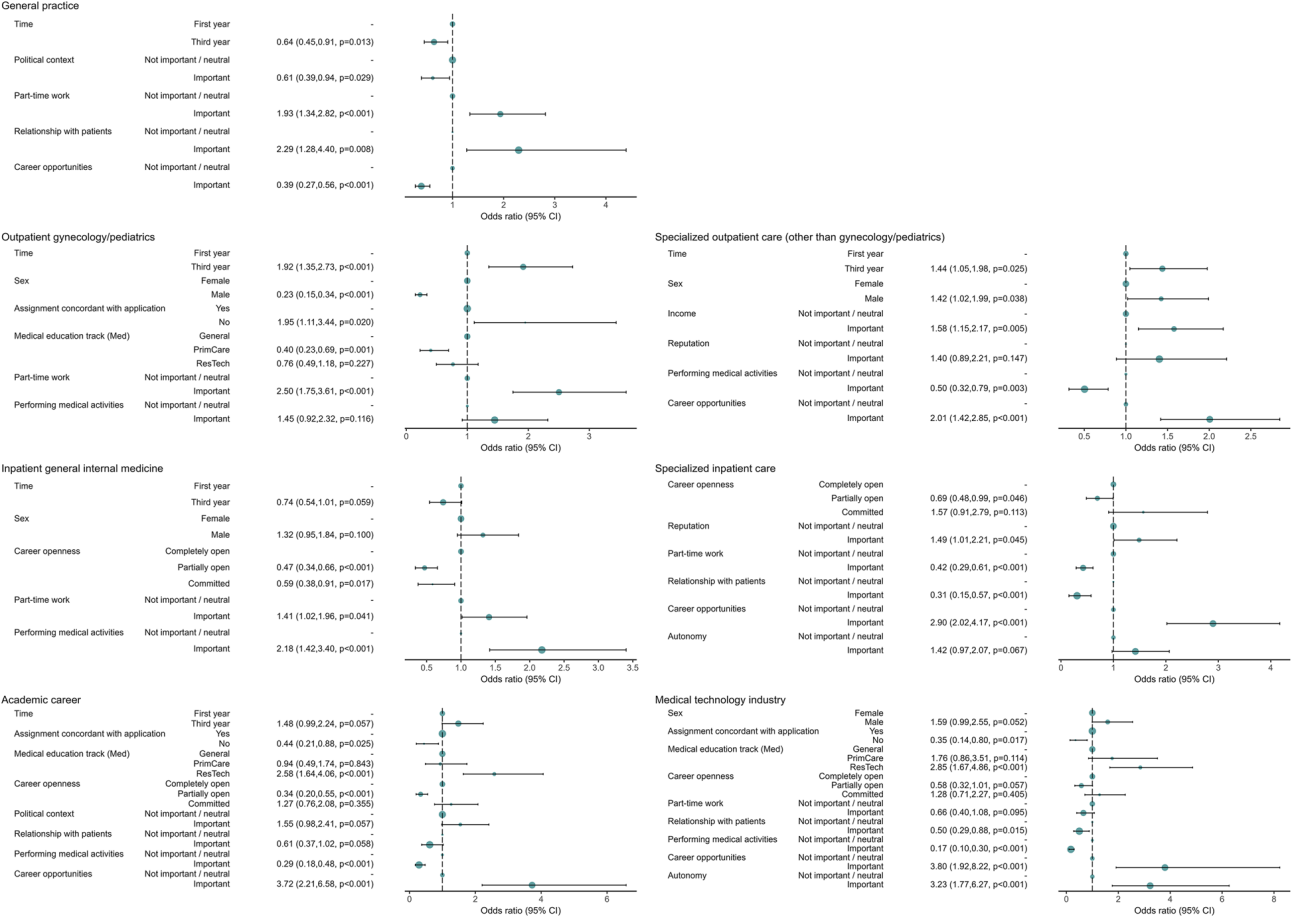
Logistic multivariable regression models (forest plot) of the attractiveness of medical career options. Odds ratios are reported with 95% confidence intervals and *p*-values. OR: odds ratio; CI: confidence interval

The trends observed in the attractiveness of each medical career option, as shown in Fig. 2, were assessed by multivariable analysis. In Fig. 5, results of the final best-performing multivariable models for the attractiveness of each career option are reported (see Additional file 1 Table 2 for univariable analysis and alternative second-best models). The adjusted analysis confirmed the positive trend in the attractiveness of outpatient gynecology/pediatrics during the bachelor’s program, OR (95% CI): 1.92 (1.35, 2.73) for a third-year student compared to a first-year student, and evidenced a positive trend also in the other specialized outpatient disciplines, 1.44 (1.05, 1.98), but a negative trend in the attractiveness of general practice, 0.64 (0.45, 0.91). In addition, there were factors associated with the attractiveness of only one or more career options. The importance of part-time work was positively associated with the attractiveness of a career in general practice, outpatient gynecology/pediatrics or inpatient general internal medicine, but negatively associated with a career in specialized inpatient care. The importance of career opportunities was positively associated with the attractiveness of a career in the specialized disciplines (outpatient and inpatient) or the research-oriented career options (academic or medical technology industry), but negatively associated with the attractiveness of general practice. Being male was positively associated with the attractiveness of a career in outpatient specialties other than gynecology/pediatrics, but negatively associated with the attractiveness of a career in outpatient gynecology/pediatrics. Rating the performance of medical activities as important was positively associated with the attractiveness of a career in inpatient general internal medicine, but negatively associated with the attractiveness of a career in outpatient specialties other than gynecology/pediatrics or in the research-oriented career options. Studying in the Med_ResTech_ track was positively associated with the attractiveness of the research-oriented career options. The importance of autonomy was only positively associated with a research-oriented career in medical technology industry. The importance of the relationship with patients and the political context were uniquely associated, positively and negatively respectively, with the attractiveness of general practice. The importance of income was only positively associated with the attractiveness of specialized outpatient care other than gynecology/pediatrics. The importance of reputation was only positively associated with the attractiveness of a career in specialized inpatient care.

## Discussion

### Summary

This study examined changes in medical students' career openness and changes in the perceived attractiveness of career options during the bachelor's program at the University of Zurich and the factors associated with these perceptions. We found that career openness decreased during the bachelor’s program, suggesting that students were already in the process of narrowing their career options. The perceived importance of medical career determinants changed significantly, with an overall increase in the importance of part-time work, autonomy and relationship with patients, and a decrease in the importance of reputation and career opportunities. Multivariable analysis showed that the importance of part-time work was positively associated with the attractiveness of general practice, outpatient gynecology/pediatrics and inpatient general internal medicine, although there was a negative trend in the attractiveness of general practice as a career option during the bachelor’s program whereas the attractiveness of outpatient specialized care increased.

### Changes in career openness

This study found that the percentage of students who were completely open to a career decreased during the bachelor’s program, but interestingly, the percentage of students who had committed to a specific medical career also decreased. As a result, there was an increase in the proportion of partially open students, i.e. students who had excluded a particular medical career as a goal without committing to any other specific career. This is consistent with previous studies showing that career intentions are rarely fixed [30–32] but that the proportion of completely undecided students decreases over time [30].

### Changes in attractiveness of medical career options and career determinants

In our study, the majority of students perceived a career in a specialized care setting (both inpatient and outpatient) as most attractive throughout the observation period and the attractiveness of outpatient gynecology/pediatrics increased significantly (mainly observed in the Med_General_ education track). These findings are consistent with the literature, although a direct comparison is not possible due to differences in the specialty definition between studies [24, 33, 34]. Moreover, longitudinal studies found that the most stable career intentions were general practice and internal medicine and the least stable were pediatrics and obstetrics-gynecology [22, 24]. In our study, one in three students appeared to be interested in a career in general practice and there was no change observed over time. Previous research found similar numbers regarding the percentage of students interested in general practice at the end of the bachelor’s program [35], but differently from our study, the attractiveness of general practice increased during the bachelor’s program. Furthermore, in our multivariable analysis, a student was less likely to be interested in general practice at the end of the bachelor’s program than at the beginning. This negative trend was also observed in another study [36].

In terms of changes in determinants of career choice, we observed a significant increase in the importance of part-time work, autonomy and relationships with patients. These factors contribute to GPs’ work satisfaction in clinical practice [37] and (except for autonomy) were associated with the attractiveness of general practice in our study. Despite the overall negative trend in the attractiveness of general practice, these determinants were rated most highly by the Med_PrimCare_ track students’, which may indicate that these students may become more interested in a career in general practice later in their medical education.

### Factors associated with the attractiveness of medical career options and career determinants

The perceived attractiveness of different career options depends on student-sided factors and the importance of determinants which we modelled using logistic regression analysis. We found that perceiving part-time as important was positively associated with the attractiveness of general practice, outpatient gynecology/pediatrics and inpatient general internal medicine, as previous studies have shown [38–40]. Consistent with our findings, many medical students who narrow their careers show a preference for part-time work, with a significantly higher proportion of female students compared to their male counterparts [9, 41–43]. In addition, our finding that being female is positively associated with the attractiveness of gynecology/pediatrics is also supported by the literature [44–49]. Moreover, we found a positive association between the importance of income and the attractiveness of specialized outpatient care, also consistent with recent findings [13]. Furthermore, students rating career opportunities as important were more likely to be attracted to specialized care (both outpatient and inpatient) or research (both academic and industry). Similar results have been found in previous studies [9, 13, 50, 51], but no previous study has analyzed research as a distinct career option. Interestingly, there were factors that were uniquely associated with the attractiveness of general practice, i.e. perceiving relationships with patients as important and perceiving the political context as unimportant. The second factor is understandable in the context of the Swiss healthcare system, where general practice is less politically regulated than other medical disciplines [52]. These results are in line with previous findings that general practice is recognized by students for long-term patient relationships and patient contact [3, 9, 13, 50, 53] and that the influence of the medico-political climate is the most negative influencing factor associated with the attractiveness of a career in general practice [53]. In addition, other factors associated with the attractiveness in general practice include the perception of independent decision-making [13], the importance of work-family balance [13, 53] and quality of life [3], better practical experiences in general practice during medical school [13] and the opinion that general practice provides a pleasant working environment [35].

### Strengths and limitations

The strengths of this study are a high response rate (71% and 87% in the first and second surveys, respectively), which represents the majority of the target population. A novelty of the study is the inclusion of research as a distinct medical career option and its relative multivariable analysis. This is important because such research-oriented students, particularly in the industrial sector, could be lost to the physician workforce, thus reducing the effectiveness of medicals schools in mitigating predicted workforce shortages.

The main limitations of this study are the anonymous nature of the data collection, which did not allow the analysis of changes in individual respondents (hence the use of a repeated cross-sectional design) and the observational design, which prevented causal inference. Furthermore, we only examined the bachelor’s program, which is half of all medical school education. As career intentions during the first three years are only moderately predictive of final medical career choices, the cohort requires further follow-up [54]. It should be noted that we observed changes in the attractiveness of medical career options during the bachelor’s program, which we were able to explain using multivariate analysis, but without capturing all confounding factors. In fact, we don’t have information about the factors that determine students’ indirect exposure to a career or the “hidden curriculum” [13, 53], such as advice, opinions or influences beyond what was formally or intentionally taught, from GPs or doctors from other specialties encountered in medical school, or advice from friends or classmates. Finally, it must be acknowledged that the educational tracks, especially Med_PrimCare_, are of limited size and, therefore, of limited power to detect small changes and differences.

### Implications for research and practice

The study is relevant to the medical education provision and then to society, as it examines the career intentions of students at the beginning and end of their medical bachelor’s studies in different educational tracks. Although it is country-specific and depends on the curricula of specific medical schools as well as on the career opportunities, healthcare and political system in Switzerland, its findings are consistent with the global trend, particularly regarding the importance of work-life balance as a career determinant [48]. This may have potential implications for promoting certain career choices, such as general practice, where there are shortages. As one in three students in our study appeared to be interested in a career in general practice at the end of the bachelor’s program, this could mean that one in ten students would definitely choose to become a GP, based on previous estimates [22, 53]. Although there is reason to believe that the attractiveness of general practice to students will increase with more direct exposure to general practice and its role model during their master’s studies [55–57], the main message of the study is to address the challenge of making primary care more attractive to students starting from the bachelor’s program. In fact, a third-year medical student was less likely to be interested in general practice compared to a first-year student. This requires special attention and further research. This might be due to an increased interest in other specialties, as the proportion of students who also found other specialties attractive was higher among third-year students than among first-year students, or to a negative perception of the general practice career or a misconception of the profession, as reported in the literature [13]. As in Switzerland GPs do not have their own specialty qualification like GPs in other European countries [58], medical students may not be aware of the specific skills and values of the profession and therefore need more guidance and support to develop positive attitudes towards the profession. For example, GP-specific perspectives and teaching during the bachelor’s program, based on case vignettes that address important determinants of general practice, such as the relationship with the patient, continuity of care, autonomy and independent decision making, and highlight that the profession allows for work-life balance and part-time work, would make students more familiar with the profession and perhaps more interested in choosing it as a career. In fact, students’ career interests are likely to be influenced by familiarity with a career and understanding of the profession’s role within the healthcare system [24]. In addition, given the evidence that societal needs influence career choices [8, 59–61], medical schools should better inform students about the reality of workforce requirements and raise awareness of the need for more general practitioners. Improving the promotion of general practice in medical schools by introducing curricular experiences of general practice, such as practice visits or placements, is known to increase attractiveness [62, 63]. Introducing early and meaningful exposure to general practice through clinical rotations and interactions with GPs could enhance the attractiveness of this career path [64] and positively influence students' career choices [65]. Long and immersive placements are even suggested and undergraduate primary care exposure should challenge students, testing not only their communication skills but also their clinical reasoning, diagnostic, ethical, and management competences [66]. In Austria an expanded four-semester curriculum for general practice within the ordinary medical school was introduced [13] to increase the orientation of graduates towards the GP profession. In Switzerland, the introduction of a specialist qualification for GPs could make general practice more attractive by giving the profession the same status as other qualifications. In addition, an adequate representation of general practice in medical schools might improve the reputation of general practice also within academic research, providing students with more research opportunities and therefore increasing their interest in the career [35].

Our results highlight the importance of part-time work, autonomy and patient contact as career determinants. Health policymakers should give priority to these aspects when undertaking measures to improve the working environment in primary care. Promoting part-time options and flexible working models could make general practice more interesting [13].

Further research, including longitudinal studies, is needed to track changes in career preferences over time and to understand the impact of early educational interventions on the attractiveness of general practice, compared with the other career options. This would help to design medical curricula that align with healthcare workforce needs. Additionally, comparative studies between different medical schools and educational tracks can identify best practices and highlight successful strategies for promoting a career in primary care.

## Conclusions

Understanding what makes a career in medicine attractive at the undergraduate level is essential for adapting medical education to the evolving needs of society and the healthcare system, particularly to address the shortage of general practitioners. During the bachelor’s program, the attractiveness of a career in general practice tended to decrease, although interest in other specialties increased. Given the increased perception of the importance of part-time work, autonomy, and patient contact as career determinants, our findings suggest that informing students about the compatibility of these determinants with a career in general practice may increase their interest in the career.

### Supplementary Information

Additional file 1: Supplementary Tables.

## Data Availability

The datasets used and/or analyzed during the current study are available from the corresponding author on reasonable request.

## Abbreviations

GP(s): General practitioner(s)
SD: Standard deviation
IQR: Interquartile range
OR: Odds ratio
CI: Confidence interval
