# Supplementary material for: Changes in the attractiveness of medical careers and career determinants during the bachelor’s program at Zurich medical schools
Source: BMC Med Educ. 2024 Jun 26;24:693. doi: 10.1186/s12909-024-05693-8 (PMC11210091; doi:10.1186/s12909-024-05693-8)
Supplement: Supplementary file 1 — Additional file 1: Supplementary Tables. [file 12909_2024_5693_MOESM1_ESM.docx]

**Supplementary Information**

**Table 1. Importance of career determinants.** Logistic multivariable regression models. OR: odds ratio. CI: confidence interval. Med_General_: medical education track without specific focus; Med_PrimCare_: medical education track with focus on primary care; Med_ResTech_ medical education track with focus on research and technology.

|  |  | Not important/  neutral | Important | OR (95% CI, p-value) |
| --- | --- | --- | --- | --- |
| Relationship with patients | | | | |
| Sex | Female | 51 (48.6) | 429 (68.4) | - |
|  | Male | 54 (51.4) | 198 (31.6) | 0.44 (0.29-0.67, p<0.001) |
| Time | First year | 60 (56.6) | 252 (39.7) | - |
|  | Third year | 46 (43.4) | 382 (60.3) | 1.93 (1.26-2.96, p=0.002) |
| Medical education track | Med_ResTech_ | 30 (28.3) | 141 (22.2) | - |
|  | Med_General_ | 63 (59.4) | 373 (58.8) | 1.11 (0.68-1.81, p=0.683) |
|  | Med_PrimCare_ | 13 (12.3) | 120 (18.9) | 1.82 (0.88-3.76, p=0.105) |
| Primarily performing medical activities | | | | |
| Sex | Female | 75 (64.1) | 405 (65.7) | - |
|  | Male | 42 (35.9) | 211 (34.3) | 0.94 (0.62-1.42, p=0.771) |
| Time | First year | 51 (42.9) | 262 (42.1) | - |
|  | Third year | 68 (57.1) | 360 (57.9) | 0.98 (0.65-1.46, p=0.906) |
| Medical education track | Med_ResTech_ | 31 (26.1) | 140 (22.5) | - |
|  | Med_General_ | 70 (58.8) | 366 (58.8) | 1.17 (0.73-1.87, p=0.511) |
|  | Med_PrimCare_ | 18 (15.1) | 116 (18.6) | 1.51 (0.79-2.87, p=0.211) |
| Autonomy | | | | |
| Sex | Female | 132 (63.5) | 348 (66.5) | - |
|  | Male | 76 (36.5) | 175 (33.5) | 0.88 (0.62-1.23, p=0.445) |
| Time | First year | 114 (54.5) | 197 (37.2) | - |
|  | Third year | 95 (45.5) | 333 (62.8) | 2.07 (1.49-2.87, p<0.001) |
| Medical education track | Med_ResTech_ | 49 (23.4) | 121 (22.8) | - |
|  | Med_General_ | 125 (59.8) | 310 (58.5) | 0.89 (0.60-1.33, p=0.573) |
|  | Med_PrimCare_ | 35 (16.7) | 99 (18.7) | 1.05 (0.63-1.78, p=0.841) |
| Part-time work | | | | |
| Sex | Female | 158 (50.3) | 322 (76.8) | - |
|  | Male | 156 (49.7) | 97 (23.2) | 0.29 (0.21-0.41, p<0.001) |
| Time | First year | 165 (52.2) | 148 (34.8) | - |
|  | Third year | 151 (47.8) | 277 (65.2) | 2.23 (1.63-3.07, p<0.001) |
| Medical education track | Med_ResTech_ | 76 (24.1) | 95 (22.4) | - |
|  | Med_General_ | 185 (58.5) | 251 (59.1) | 0.92 (0.63-1.35, p=0.668) |
|  | Med_PrimCare_ | 55 (17.4) | 79 (18.6) | 0.97 (0.60-1.58, p=0.907) |
| Career opportunities | | | | |
| Sex | Female | 170 (78.0) | 310 (60.2) | - |
|  | Male | 48 (22.0) | 205 (39.8) | 2.35 (1.62-3.41, p<0.001) |
| Time | First year | 65 (29.4) | 248 (47.7) | - |
|  | Third year | 156 (70.6) | 272 (52.3) | 0.46 (0.33-0.65, p<0.001) |
| Medical education track | Med_ResTech_ | 37 (16.7) | 134 (25.8) | - |
|  | Med_General_ | 141 (63.8) | 295 (56.7) | 0.67 (0.43-1.02, p=0.063) |
|  | Med_PrimCare_ | 43 (19.5) | 91 (17.5) | 0.65 (0.38-1.10, p=0.111) |
| Income | | | | |
| Sex | Female | 271 (67.2) | 209 (63.3) | - |
|  | Male | 132 (32.8) | 121 (36.7) | 1.21 (0.89-1.65, p=0.222) |
| Time | First year | 171 (42.1) | 142 (42.4) | - |
|  | Third year | 235 (57.9) | 193 (57.6) | 0.96 (0.71-1.29, p=0.780) |
| Medical education track | Med_ResTech_ | 109 (26.8) | 62 (18.5) | - |
|  | Med_General_ | 227 (55.9) | 209 (62.4) | 1.62 (1.12-2.34, p=0.010) |
|  | Med_PrimCare_ | 70 (17.2) | 64 (19.1) | 1.57 (0.99-2.51, p=0.057) |
| Reputation | | | | |
| Sex | Female | 344 (70.1) | 136 (56.2) | - |
|  | Male | 147 (29.9) | 106 (43.8) | 1.83 (1.32-2.53, p<0.001) |
| Time | First year | 179 (36.2) | 133 (54.3) | - |
|  | Third year | 316 (63.8) | 112 (45.7) | 0.48 (0.35-0.66, p<0.001) |
| Medical education track | Med_ResTech_ | 105 (21.2) | 66 (26.9) | - |
|  | Med_General_ | 298 (60.2) | 138 (56.3) | 0.81 (0.55-1.18, p=0.275) |
|  | Med_PrimCare_ | 92 (18.6) | 41 (16.7) | 0.81 (0.50-1.33, p=0.411) |
| Political context | | | | |
| Sex | Female | 372 (64.4) | 107 (69.5) | - |
|  | Male | 206 (35.6) | 47 (30.5) | 0.78 (0.53-1.15, p=0.210) |
| Time | First year | 246 (42.2) | 65 (41.7) | - |
|  | Third year | 337 (57.8) | 91 (58.3) | 1.07 (0.75-1.55, p=0.703) |
| Medical education track | Med_ResTech_ | 129 (22.1) | 41 (26.3) | - |
|  | Med_General_ | 344 (59.0) | 92 (59.0) | 0.82 (0.53-1.25, p=0.353) |
|  | Med_PrimCare_ | 110 (18.9) | 23 (14.7) | 0.61 (0.34-1.09, p=0.093) |

**Table 2. Attractiveness of medical career options.** Logistic regression models univariable and multivariable. Multivariable models included selected predictors using stepwise analysis but forcing sex and year to stay in the model. Results of the final best models are shown in Figure 5 (main manuscript).

Assignment concordant with application: case in which the student was assigned to the educational track applied for. OR: odds ratio. CI: confidence interval.

| General practice | | | | | |
| --- | --- | --- | --- | --- | --- |
|  |  | Not attractive/  neutral | Attractive | Univariable | Multivariable |
|  |  |  |  | OR (95% CI, p-value) | OR (95% CI, p-value) |
| Sex | Female | 335 (63.9) | 145 (69.4) | - | - |
|  | Male | 189 (36.1) | 64 (30.6) | 0.78 (0.55-1.10, p=0.162) | 1.22 (0.83-1.79, p=0.313) |
| Time | First year | 223 (41.9) | 95 (44.2) | - | - |
|  | Third year | 309 (58.1) | 120 (55.8) | 0.91 (0.66-1.25, p=0.570) | 0.64 (0.44-0.91, p=0.013) |
| Aptitude test score | Mean (SD) | 88.0 (9.1) | 87.3 (13.8) | 0.99 (0.98-1.01, p=0.511) | - |
| Assignment concordant with application N(%) | Yes | 462 (86.8) | 180 (83.7) | - | - |
|  | No | 70 (13.2) | 35 (16.3) | 1.28 (0.83-1.99, p=0.267) | - |
| Career openness | Completely open | 254 (47.7) | 102 (47.4) | - | - |
|  | Partially open | 193 (36.3) | 83 (38.6) | 1.07 (0.76-1.51, p=0.697) | - |
|  | Committed | 85 (16.0) | 30 (14.0) | 0.88 (0.55-1.41, p=0.595) | - |
| Medical Education Track | Med_General_ | 315 (59.2) | 123 (57.2) | - | - |
|  | Med_PrimCare_ | 94 (17.7) | 42 (19.5) | 1.14 (0.75-1.74, p=0.529) | - |
|  | Med_ResTech_ | 123 (23.1) | 50 (23.3) | 1.04 (0.71-1.54, p=0.839) | - |
| Income | Not Important/neutral | 275 (52.1) | 131 (61.5) | - | - |
|  | Important | 253 (47.9) | 82 (38.5) | 0.68 (0.49-0.94, p=0.020) | - |
| Reputation | Not Important/neutral | 336 (63.8) | 159 (74.6) | - | - |
|  | Important | 191 (36.2) | 54 (25.4) | 0.60 (0.42-0.85, p=0.005) | - |
| Political context | Not Important/neutral | 403 (76.6) | 180 (84.5) | - | - |
|  | Important | 123 (23.4) | 33 (15.5) | 0.60 (0.39-0.92, p=0.018) | 0.60 (0.39-0.94, p=0.025) |
| Part-time work | Not Important/neutral | 256 (48.5) | 60 (28.2) | - | - |
|  | Important | 272 (51.5) | 153 (71.8) | 2.40 (1.70-3.39, p<0.001) | 2.03 (1.38-2.98, p<0.001) |
| Relationship with patients | Not Important/neutral | 92 (17.5) | 14 (6.6) | - | - |
|  | Important | 435 (82.5) | 199 (93.4) | 3.01 (1.67-5.41, p<0.001) | 2.55 (1.35-4.82, p=0.004) |
| Performing medical activities | Not Important/neutral | 86 (16.3) | 33 (15.5) | - | - |
|  | Important | 442 (83.7) | 180 (84.5) | 1.06 (0.69-1.64, p=0.790) | - |
| Career opportunities | Not Important/neutral | 122 (23.1) | 99 (46.5) | - | - |
|  | Important | 406 (76.9) | 114 (53.5) | 0.35 (0.25-0.48, p<0.001) | 0.38 (0.26-0.55, p<0.001) |
| Autonomy | Not Important/neutral | 145 (27.5) | 64 (30.2) | - | - |
|  | Important | 382 (72.5) | 148 (69.8) | 0.88 (0.62-1.25, p=0.465) | - |

| Outpatient ginecology/pediatrics | | | | | |
| --- | --- | --- | --- | --- | --- |
|  |  | Not attractive/  neutral | Attractive | Univariable | Multivariable |
|  |  |  |  | OR (95% CI, p-value) | OR (95% CI, p-value) |
| Sex | Female | 250 (53.6) | 230 (86.1) | - | - |
|  | Male | 216 (46.4) | 37 (13.9) | 0.19 (0.13-0.28, p<0.001) | 0.23 (0.15-0.34, p<0.001) |
| Time | First year | 231 (48.7) | 87 (31.9) | - | - |
|  | Third year | 243 (51.3) | 186 (68.1) | 2.03 (1.49-2.78, p<0.001) | 1.92 (1.35-2.72, p<0.001) |
| Aptitude test score | Mean (SD) | 87.6 (9.7) | 88.1 (12.1) | 1.01 (0.99-1.02, p=0.537) | - |
| Assignment concordant with application N(%) | Yes | 414 (87.3) | 228 (83.5) | - | - |
|  | No | 60 (12.7) | 45 (16.5) | 1.36 (0.90-2.07, p=0.149) | 1.95 (1.11-3.43, p=0.020) |
| Career openness | Completely open | 239 (50.4) | 117 (42.9) | - | - |
|  | Partially open | 159 (33.5) | 117 (42.9) | 1.50 (1.09-2.08, p=0.014) | - |
|  | Committed | 76 (16.0) | 39 (14.3) | 1.05 (0.67-1.64, p=0.836) | - |
| Medical Education Track | Med_General_ | 263 (55.5) | 175 (64.1) | - | - |
|  | Med_PrimCare_ | 95 (20.0) | 41 (15.0) | 0.65 (0.43-0.98, p=0.040) | 0.40 (0.23-0.70, p=0.001) |
|  | Med_ResTech_ | 116 (24.5) | 57 (20.9) | 0.74 (0.51-1.07, p=0.108) | 0.76 (0.50-1.18, p=0.227) |
| Income | Not Important/neutral | 249 (53.0) | 157 (57.9) | - | - |
|  | Important | 221 (47.0) | 114 (42.1) | 0.82 (0.61-1.11, p=0.192) | - |
| Reputation | Not Important/neutral | 292 (62.1) | 203 (75.2) | - | - |
|  | Important | 178 (37.9) | 67 (24.8) | 0.54 (0.39-0.76, p<0.001) | - |
| Political context | Not Important/neutral | 377 (80.4) | 206 (76.3) | - | - |
|  | Important | 92 (19.6) | 64 (23.7) | 1.27 (0.89-1.83, p=0.190) | - |
| Part-time work | Not Important/neutral | 250 (53.2) | 66 (24.4) | - | - |
|  | Important | 220 (46.8) | 205 (75.6) | 3.53 (2.53-4.92, p<0.001) | 2.50 (1.74-3.59, p<0.001) |
| Relationship with patients | Not Important/neutral | 86 (18.3) | 20 (7.4) | - | - |
|  | Important | 383 (81.7) | 251 (92.6) | 2.82 (1.69-4.70, p<0.001) | - |
| Performing medical activities | Not Important/neutral | 82 (17.4) | 37 (13.7) | - | - |
|  | Important | 388 (82.6) | 234 (86.3) | 1.34 (0.88-2.04, p=0.177) | 1.45 (0.91-2.30, p=0.116) |
| Career opportunities | Not Important/neutral | 116 (24.7) | 105 (38.7) | - | - |
|  | Important | 354 (75.3) | 166 (61.3) | 0.52 (0.38-0.71, p<0.001) | - |
| Autonomy | Not Important/neutral | 133 (28.4) | 76 (28.1) | - | - |
|  | Important | 336 (71.6) | 194 (71.9) | 1.01 (0.72-1.41, p=0.951) | - |

| Specialized outpatient care (other than gynecology/pediatrics) | | | | | |
| --- | --- | --- | --- | --- | --- |
|  |  | Not attractive/  neutral | Attractive | Univariable | Multivariable |
|  |  |  |  | OR (95% CI, p-value) | OR (95% CI, p-value) |
| Sex | Female | 207 (71.4) | 273 (61.6) | - | - |
|  | Male | 83 (28.6) | 170 (38.4) | 1.55 (1.13-2.14, p=0.007) | 1.42 (1.02-1.99, p=0.038) |
| Time | First year | 133 (45.4) | 185 (40.7) | - | - |
|  | Third year | 160 (54.6) | 269 (59.3) | 1.21 (0.90-1.63, p=0.210) | 1.44 (1.05-1.97, p=0.025) |
| Aptitude test score | Mean (SD) | 87.9 (10.5) | 87.7 (10.7) | 1.00 (0.98-1.01, p=0.785) | - |
| Assignment concordant with application N(%) | Yes | 254 (86.7) | 388 (85.5) | - | - |
|  | No | 39 (13.3) | 66 (14.5) | 1.11 (0.72-1.70, p=0.638) | - |
| Career openness | Completely open | 137 (46.8) | 219 (48.2) | - | - |
|  | Partially open | 112 (38.2) | 164 (36.1) | 0.92 (0.66-1.26, p=0.593) | - |
|  | Committed | 44 (15.0) | 71 (15.6) | 1.01 (0.66-1.56, p=0.966) | - |
| Medical Education Track | Med_General_ | 173 (59.0) | 265 (58.4) | - | - |
|  | Med_PrimCare_ | 53 (18.1) | 83 (18.3) | 1.02 (0.69-1.52, p=0.912) | - |
|  | Med_ResTech_ | 67 (22.9) | 106 (23.3) | 1.03 (0.72-1.48, p=0.861) | - |
| Income | Not Important/neutral | 185 (63.6) | 221 (49.1) | - | - |
|  | Important | 106 (36.4) | 229 (50.9) | 1.81 (1.34-2.45, p<0.001) | 1.58 (1.15-2.17, p=0.005) |
| Reputation | Not Important/neutral | 213 (73.2) | 282 (62.8) | - | - |
|  | Important | 78 (26.8) | 167 (37.2) | 1.62 (1.17-2.23, p=0.003) | - |
| Political context | Not Important/neutral | 231 (79.4) | 352 (78.6) | - | - |
|  | Important | 60 (20.6) | 96 (21.4) | 1.05 (0.73-1.51, p=0.792) | - |
| Part-time work | Not Important/neutral | 120 (41.2) | 196 (43.6) | - | - |
|  | Important | 171 (58.8) | 254 (56.4) | 0.91 (0.67-1.23, p=0.533) | - |
| Relationship with patients | Not Important/neutral | 42 (14.4) | 64 (14.3) | - | - |
|  | Important | 249 (85.6) | 385 (85.7) | 1.01 (0.67-1.55, p=0.946) | 1.40 (0.89-2.21, p=0.147) |
| Performing medical activities | Not Important/neutral | 34 (11.7) | 85 (18.9) | - | - |
|  | Important | 257 (88.3) | 365 (81.1) | 0.57 (0.37-0.87, p=0.010) | 0.50 (0.32-0.79, p=0.003) |
| Career opportunities | Not Important/neutral | 113 (38.8) | 108 (24.0) | - | - |
|  | Important | 178 (61.2) | 342 (76.0) | 2.01 (1.46-2.77, p<0.001) | 2.01 (1.42-2.84, p<0.001) |
| Autonomy | Not Important/neutral | 96 (33.0) | 113 (25.2) | - | - |
|  | Important | 195 (67.0) | 335 (74.8) | 1.46 (1.06-2.02, p=0.022) | - |

| Inpatient general internal medicine | | | | | |
| --- | --- | --- | --- | --- | --- |
|  |  | Not attractive/  neutral | Attractive | Univariable | Multivariable |
|  |  |  |  | OR (95% CI, p-value) | OR (95% CI, p-value) |
| Sex | Female | 279 (67.6) | 201 (62.8) | - | - |
|  | Male | 134 (32.4) | 119 (37.2) | 1.23 (0.91-1.67, p=0.181) | 1.32 (0.95-1.84, p=0.100) |
| Time | First year | 166 (39.3) | 152 (46.8) | - | - |
|  | Third year | 256 (60.7) | 173 (53.2) | 0.74 (0.55-0.99, p=0.042) | 0.74 (0.54-1.01, p=0.059) |
| Aptitude test score | Mean (SD) | 87.6 (11.7) | 88.0 (9.2) | 1.00 (0.99-1.02, p=0.674) | - |
| Assignment concordant with application N(%) | Yes | 363 (86.0) | 279 (85.8) | - | - |
|  | No | 59 (14.0) | 46 (14.2) | 1.01 (0.67-1.54, p=0.946) | - |
| Career openness | Completely open | 169 (40.0) | 187 (57.5) | - | - |
|  | Partially open | 183 (43.4) | 93 (28.6) | 0.46 (0.33-0.64, p<0.001) | 0.47 (0.34-0.66, p<0.001) |
|  | Committed | 70 (16.6) | 45 (13.8) | 0.58 (0.38-0.89, p=0.013) | 0.59 (0.38-0.91, p=0.017) |
| Medical Education Track | Med_General_ | 258 (61.1) | 180 (55.4) | - | - |
|  | Med_PrimCare_ | 72 (17.1) | 64 (19.7) | 1.27 (0.87-1.88, p=0.220) | - |
|  | Med_ResTech_ | 92 (21.8) | 81 (24.9) | 1.26 (0.89-1.80, p=0.198) | - |
| Income | Not Important/neutral | 223 (53.2) | 183 (56.8) | - | - |
|  | Important | 196 (46.8) | 139 (43.2) | 0.86 (0.65-1.16, p=0.328) | - |
| Reputation | Not Important/neutral | 284 (67.9) | 211 (65.5) | - | - |
|  | Important | 134 (32.1) | 111 (34.5) | 1.11 (0.82-1.52, p=0.489) | - |
| Political context | Not Important/neutral | 328 (78.5) | 255 (79.4) | - | - |
|  | Important | 90 (21.5) | 66 (20.6) | 0.94 (0.66-1.35, p=0.749) | - |
| Part-time work | Not Important/neutral | 187 (44.6) | 129 (40.1) | - | - |
|  | Important | 232 (55.4) | 193 (59.9) | 1.21 (0.90-1.62, p=0.213) | 1.41 (1.01-1.96, p=0.041) |
| Relationship with patients | Not Important/neutral | 64 (15.3) | 42 (13.0) | - | - |
|  | Important | 354 (84.7) | 280 (87.0) | 1.21 (0.79-1.83, p=0.383) | - |
| Performing medical activities | Not Important/neutral | 82 (19.6) | 37 (11.5) | - | - |
|  | Important | 337 (80.4) | 285 (88.5) | 1.87 (1.23-2.85, p=0.003) | 2.18 (1.41-3.37, p<0.001) |
| Career opportunities | Not Important/neutral | 131 (31.3) | 90 (28.0) | - | - |
|  | Important | 288 (68.7) | 232 (72.0) | 1.17 (0.85-1.61, p=0.328) | - |
| Autonomy | Not Important/neutral | 114 (27.3) | 95 (29.6) | - | - |
|  | Important | 304 (72.7) | 226 (70.4) | 0.89 (0.65-1.23, p=0.487) | - |

| Inpatient specialized care | | | | | |
| --- | --- | --- | --- | --- | --- |
|  |  | Not attractive/  neutral | Attractive | Univariable | Multivariable |
|  |  |  |  | OR (95% CI, p-value) | OR (95% CI, p-value) |
| Sex | Female | 189 (74.4) | 291 (60.8) | - | - |
|  | Male | 65 (25.6) | 188 (39.2) | 1.88 (1.34-2.63, p<0.001) | 1.07 (0.73-1.58, p=0.716) |
| Time | First year | 92 (35.5) | 226 (46.3) | - | - |
|  | Third year | 167 (64.5) | 262 (53.7) | 0.64 (0.47-0.87, p=0.005) | 1.02 (0.71-1.48, p=0.907) |
| Aptitude test score | Mean (SD) | 87.9 (12.3) | 87.8 (9.7) | 1.00 (0.98-1.02, p=0.911) | - |
| Assignment concordant with application N(%) | Yes | 219 (84.6) | 423 (86.7) | - | - |
|  | No | 40 (15.4) | 65 (13.3) | 0.84 (0.55-1.29, p=0.427) | - |
| Career openness | Completely open | 120 (46.3) | 236 (48.4) | - | - |
|  | Partially open | 117 (45.2) | 159 (32.6) | 0.69 (0.50-0.96, p=0.026) | 0.66 (0.46-0.96, p=0.028) |
|  | Committed | 22 (8.5) | 93 (19.1) | 2.15 (1.29-3.59, p=0.004) | 1.54 (0.88-2.69, p=0.134) |
| Medical Education Track | Med_General_ | 165 (63.7) | 273 (55.9) | - | - |
|  | Med_PrimCare_ | 49 (18.9) | 87 (17.8) | 1.07 (0.72-1.60, p=0.729) | - |
|  | Med_ResTech_ | 45 (17.4) | 128 (26.2) | 1.72 (1.16-2.54, p=0.007) | - |
| Income | Not Important/neutral | 159 (61.9) | 247 (51.0) | - | - |
|  | Important | 98 (38.1) | 237 (49.0) | 1.56 (1.14-2.12, p=0.005) | - |
| Reputation | Not Important/neutral | 203 (79.0) | 292 (60.5) | - | - |
|  | Important | 54 (21.0) | 191 (39.5) | 2.46 (1.73-3.49, p<0.001) | 1.53 (1.02-2.28, p=0.038) |
| Political context | Not Important/neutral | 211 (82.1) | 372 (77.2) | - | - |
|  | Important | 46 (17.9) | 110 (22.8) | 1.36 (0.92-1.99, p=0.119) | - |
| Part-time work | Not Important/neutral | 62 (24.1) | 254 (52.5) | - | - |
|  | Important | 195 (75.9) | 230 (47.5) | 0.29 (0.21-0.40, p<0.001) | 0.42 (0.29-0.62, p<0.001) |
| Relationship with patients | Not Important/neutral | 12 (4.7) | 94 (19.5) | - | - |
|  | Important | 245 (95.3) | 389 (80.5) | 0.20 (0.11-0.38, p<0.001) | 0.28 (0.14-0.56, p<0.001) |
| Performing medical activities | Not Important/neutral | 44 (17.1) | 75 (15.5) | - | - |
|  | Important | 213 (82.9) | 409 (84.5) | 1.13 (0.75-1.69, p=0.567) | - |
| Career opportunities | Not Important/neutral | 129 (50.2) | 92 (19.0) | - | - |
|  | Important | 128 (49.8) | 392 (81.0) | 4.29 (3.08-6.00, p<0.001) | 2.85 (1.97-4.13, p<0.001) |
| Autonomy | Not Important/neutral | 86 (33.5) | 123 (25.5) | - | - |
|  | Important | 171 (66.5) | 359 (74.5) | 1.47 (1.06-2.04, p=0.023) | 1.38 (0.94-2.03, p=0.099) |

| Academic career | | | | | |
| --- | --- | --- | --- | --- | --- |
|  |  | Not attractive/ | Attractive | Univariable | Multivariable |
|  |  | neutral |  | OR (95% CI, p-value) | OR (95% CI, p-value) |
| Sex | Female | 387 (67.3) | 93 (58.9) | - | - |
|  | Male | 188 (32.7) | 65 (41.1) | 1.44 (1.00-2.07, p=0.049) | 1.02 (0.68-1.54, p=0.925) |
| Time | First year | 249 (42.3) | 69 (43.4) | - | - |
|  | Third year | 339 (57.7) | 90 (56.6) | 0.96 (0.67-1.36, p=0.812) | 1.49 (0.99-2.24, p=0.057) |
| Aptitude test score | Mean (SD) | 87.4 (10.5) | 89.1 (11.3) | 1.02 (1.00-1.04, p=0.103) | - |
| Assignment concordant with application N(%) | Yes | 496 (84.4) | 146 (91.8) | - | - |
|  | No | 92 (15.6) | 13 (8.2) | 0.48 (0.26-0.88, p=0.018) | 0.43 (0.21-0.88, p=0.021) |
| Career openness | Completely open | 263 (44.7) | 93 (58.5) | - | - |
|  | Partially open | 249 (42.3) | 27 (17.0) | 0.31 (0.19-0.49, p<0.001) | 0.34 (0.21-0.57, p<0.001) |
|  | Committed | 76 (12.9) | 39 (24.5) | 1.45 (0.92-2.28, p=0.107) | 1.25 (0.76-2.07, p=0.379) |
| Medical Education Track | Med_General_ | 359 (61.1) | 79 (49.7) | - | - |
|  | Med_PrimCare_ | 118 (20.1) | 18 (11.3) | 0.69 (0.40-1.20, p=0.194) | 1.00 (0.53-1.89, p=0.999) |
|  | Med_ResTech_ | 111 (18.9) | 62 (39.0) | 2.54 (1.71-3.77, p<0.001) | 2.64 (1.67-4.17, p<0.001) |
| Income | Not Important/neutral | 327 (56.2) | 79 (49.7) | - | - |
|  | Important | 255 (43.8) | 80 (50.3) | 1.30 (0.91-1.85, p=0.145) | - |
| Reputation | Not Important/neutral | 401 (69.0) | 94 (59.1) | - | - |
|  | Important | 180 (31.0) | 65 (40.9) | 1.54 (1.07-2.21, p=0.019) | - |
| Political context | Not Important/neutral | 470 (81.0) | 113 (71.1) | - | - |
|  | Important | 110 (19.0) | 46 (28.9) | 1.74 (1.17-2.60, p=0.007) | 1.60 (1.02-2.52, p=0.042) |
| Part-time work | Not Important/neutral | 236 (40.5) | 80 (50.3) | - | - |
|  | Important | 346 (59.5) | 79 (49.7) | 0.67 (0.47-0.96, p=0.028) | - |
| Relationship with patients | Not Important/neutral | 66 (11.4) | 40 (25.2) | - | - |
|  | Important | 515 (88.6) | 119 (74.8) | 0.38 (0.25-0.59, p<0.001) | 0.60 (0.36-0.99, p=0.046) |
| Performing medical activities | Not Important/neutral | 71 (12.2) | 48 (30.2) | - | - |
|  | Important | 511 (87.8) | 111 (69.8) | 0.32 (0.21-0.49, p<0.001) | 0.30 (0.18-0.48, p<0.001) |
| Career opportunities | Not Important/neutral | 201 (34.5) | 20 (12.6) | - | - |
|  | Important | 381 (65.5) | 139 (87.4) | 3.67 (2.23-6.04, p<0.001) | 3.93 (2.25-6.87, p<0.001) |
| Autonomy | Not Important/neutral | 173 (29.8) | 36 (22.6) | - | - |
|  | Important | 407 (70.2) | 123 (77.4) | 1.45 (0.96-2.19, p=0.076) | - |

| Medical technology industry research | | | | | |
| --- | --- | --- | --- | --- | --- |
|  |  | Not attractive/  neutral | Attractive | Univariable | Multivariable |
|  |  |  |  | OR (95% CI, p-value) | OR (95% CI, p-value) |
| Sex | Female | 424 (68.4) | 56 (49.6) | - | - |
|  | Male | 196 (31.6) | 57 (50.4) | 2.20 (1.47-3.30, p<0.001) | 1.59 (0.99-2.55, p=0.054) |
| Time | First year | 265 (41.9) | 53 (46.5) | - | - |
|  | Third year | 368 (58.1) | 61 (53.5) | 0.83 (0.56-1.24, p=0.358) | 1.02 (0.63-1.66, p=0.920) |
| Aptitude test score | Mean (SD) | 87.5 (11.0) | 89.2 (8.7) | 1.02 (0.99-1.04, p=0.151) | - |
| Assignment concordant with application N(%) | Yes | 537 (84.8) | 105 (92.1) | - | - |
|  | No | 96 (15.2) | 9 (7.9) | 0.48 (0.23-0.98, p=0.044) | 0.35 (0.15-0.83, p=0.017) |
| Career openness | Completely open | 295 (46.6) | 61 (53.5) | - | - |
|  | Partially open | 252 (39.8) | 24 (21.1) | 0.46 (0.28-0.76, p=0.002) | 0.58 (0.33-1.02, p=0.057) |
|  | Committed | 86 (13.6) | 29 (25.4) | 1.63 (0.99-2.70, p=0.057) | 1.28 (0.72-2.28, p=0.409) |
| Medical Education Track | Med_General_ | 385 (60.8) | 53 (46.5) | - | - |
|  | Med_PrimCare_ | 120 (19.0) | 16 (14.0) | 0.97 (0.53-1.76, p=0.916) | 1.76 (0.87-3.56, p=0.116) |
|  | Med_ResTech_ | 128 (20.2) | 45 (39.5) | 2.55 (1.64-3.98, p<0.001) | 2.86 (1.67-4.89, p<0.001) |
| Income | Not Important/neutral | 358 (57.1) | 48 (42.1) | - | - |
|  | Important | 269 (42.9) | 66 (57.9) | 1.83 (1.22-2.74, p=0.003) | - |
| Reputation | Not Important/neutral | 433 (69.2) | 62 (54.4) | - | - |
|  | Important | 193 (30.8) | 52 (45.6) | 1.88 (1.25-2.82, p=0.002) | - |
| Political context | Not Important/neutral | 498 (79.7) | 85 (74.6) | - | - |
|  | Important | 127 (20.3) | 29 (25.4) | 1.34 (0.84-2.13, p=0.219) | - |
| Part-time work | Not Important/neutral | 253 (40.4) | 63 (55.3) | - | - |
|  | Important | 374 (59.6) | 51 (44.7) | 0.55 (0.37-0.82, p=0.003) | 0.65 (0.40-1.08, p=0.095) |
| Relationship with patients | Not Important/neutral | 70 (11.2) | 36 (31.6) | - | - |
|  | Important | 556 (88.8) | 78 (68.4) | 0.27 (0.17-0.43, p<0.001) | 0.50 (0.28-0.87, p=0.015) |
| Performing medical activities | Not Important/neutral | 76 (12.1) | 43 (37.7) | - | - |
|  | Important | 551 (87.9) | 71 (62.3) | 0.23 (0.15-0.36, p<0.001) | 0.17 (0.10-0.30, p<0.001) |
| Career opportunities | Not Important/neutral | 209 (33.3) | 12 (10.5) | - | - |
|  | Important | 418 (66.7) | 102 (89.5) | 4.25 (2.28-7.91, p<0.001) | 3.80 (1.85-7.84, p<0.001) |
| Autonomy | Not Important/neutral | 194 (31.0) | 15 (13.2) | - | - |
|  | Important | 431 (69.0) | 99 (86.8) | 2.97 (1.68-5.25, p<0.001) | 3.21 (1.70-6.07, p<0.001) |
